# Host traits rather than migration and molting strategies explain feather bacterial load in Palearctic passerines

**DOI:** 10.1016/j.isci.2024.111079

**Published:** 2024-09-30

**Authors:** Veronika Gvoždíková Javůrková, Vojtěch Brlík, Petr Heneberg, Milica Požgayová, Petr Procházka, Maurine W. Dietz, Joana Falcao Salles, B. Irene Tieleman

**Affiliations:** 1 Groningen Institute for Evolutionary Life Sciences, University of Groningen, Nijenborgh 7, 9747 AG Groningen, the Netherlands; 2 Institute of Vertebrate Biology of the Czech Academy of Sciences, Květná 8, 603 65 Brno, Czech Republic; 3 Faculty of Sciences, Charles University, Department of Ecology, Viničná 7, 128 44 Prague, Czech Republic; 4 Third Faculty of Medicine, Charles University, Ruská 87, 100 00 Prague, Czech Republic

**Keywords:** Ornithology, Microbiology, Evolutionary biology

## Abstract

Feather bacterial load affects key avian life-history traits such as plumage condition, innate immunity, and reproductive success. Investigating the interplay between life-history traits and feather microbial load is critical for understanding mechanisms of host-microbiome interactions. We hypothesize that spatiotemporal variation associated with migration and molting, body size affecting colonizable body surface area, and preening intensity could shape feather bacterial load. Integrating 16S rDNA-qPCR and flow cytometry, we examined total and viable bacterial loads in the feathers of 316 individuals of 24 Palearctic passerine species. We found that viable bacterial load in feathers was lower in larger species and higher in residents compared to migrants. In contrast, total bacterial load was not explained by any of the life-history traits but varied considerably among species, sampling sites, and years. By pinpointing main drivers of bacterial loads on avian body surfaces, we identify key mechanisms shaping host-microbiome interactions and open alternative research directions.

## Highlights

• Viable feather bacterial load is lower in larger species of Palearctic passerines
• Resident species have higher viable feather bacterial loads than migrants
• Total feather bacterial load is not affected by migratory or molt strategies
• Species, sampling site, and year explain most of the variation in feather bacterial load

## Graphical abstract

**Figure:**
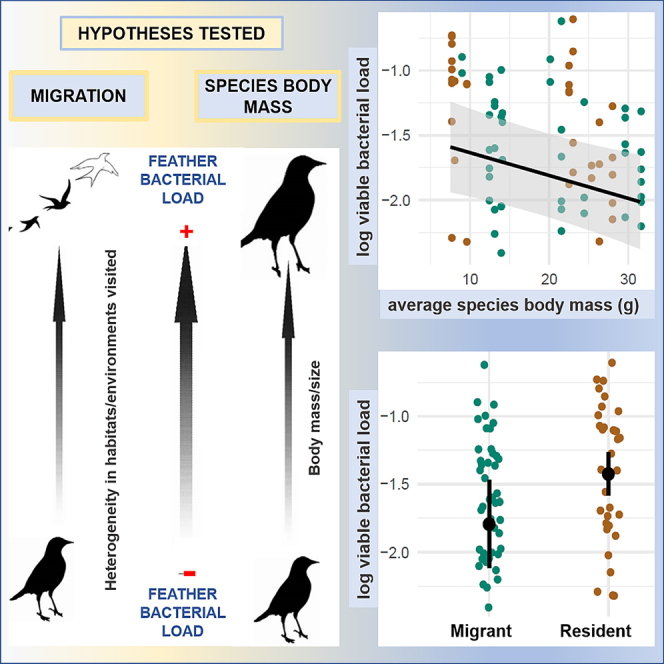

## Introduction

Feathers are an indispensable part of the bird’s body providing essential functions such as flight ability,^1^ thermoregulation,^2^ and sexual signaling.^3^^,^^4^ Feathers, as the uppermost integument layer, are also in continuous contact with the environment and the microorganisms living therein. Despite the intense contact of birds with a wide variety of environmental microorganisms,^5^^,^^6^ the microbial communities on feathers are strongly species specific^7^^,^^8^ and may serve different functions. For example, important microbial species found on feathers are antimicrobial-producing bacteria that maintain species-specific feather microbiomes and bacteria capable of producing volatile chemosignals used for olfactory communication in avian hosts.^9^^,^^10^^,^^11^^,^^12^^,^^13^ Not only the diversity and structure of the feather microbiome but also the microbial abundance on feathers may be related to life-history traits in birds. The bacterial load on feathers has been shown to vary among species^14^ and to influence immune function,^15^^,^^16^ reproductive success expressed as the number of successful fledglings,^17^^,^^18^ and overall feather quality,^19^^,^^20^^,^^21^^,^^22^ most likely via alterations in the energy effort invested in preening and feather maintenance. While the mechanisms that maintain the species-specific feather microbiome remain poorly understood, horizontal microbial acquisition from the environment appears to be essential for feather microbiome assembly as suggested by previous studies showing similarity between feather and habitat- or nest-associated bacterial communities.^7^^,^^8^^,^^23^^,^^24^ In addition, the microbiome of the animal’s integument is subject to the animal’s dynamic changes over time, with several studies showing the effects of sloughing in amphibians or molt in cetaceans in shaping both the diversity and abundance of the skin microbiome.^25^^,^^26^ It is, therefore, likely that avian life-history traits, such as seasonal migration and timing of feather replacement, which result in differences in the duration of exposure to the environment, may explain interspecific variability in the feather microbial abundance in birds.

Migration strategy affects the diversity of the environments occupied throughout the annual cycle, which in turn may alter the processes of feather microbiome acquisition. Migratory birds are exposed to heterogeneous environments while moving between breeding and non-breeding grounds, which could enrich the microbial communities in their feathers. In line with this hypothesis, migratory birds within the Nearctic-Neotropical flyway were found to carry more diverse microbial communities than temperate and tropical residents in the region.^27^ Similarly, most of the feather microbial community of common swifts (*Apus apus*) was composed of microorganisms acquired during migration rather than during the stationary breeding period.^28^

Molt strategy affects the timing of feather replacement and, thus, the exposure of feathers to environmental microorganisms. Resident passerines in temperate regions typically molt after breeding, whereas long-distance migrants within the Palearctic show a range of molting strategies.^29^ Migratory species may molt after breeding, during the non-breeding period, or even during both periods.^30^ The exposure of feathers to environmental microbes thus varies greatly between species with different molting strategies with potential implications for microbial loads on feathers. In addition, both migration^31^ and molting^32^^,^^33^ are energetically demanding processes that may alter the investment in feather self-maintenance, including preening,^34^^,^^35^ which has been shown to reduce feather microbial loads.^19^^,^^36^^,^^37^^,^^38^ Furthermore, previous studies documented a relationship between preen gland size and density of specific feather bacteria.^37^^,^^39^^,^^40^^,^^41^ As this relationship has previously been documented in an urbanizing context,^37^ and for colonially breeding species,^39^^,^^40^ it is unknown how variability in preen gland volume, which is a proxy for preen gland secretory potential,^42^ might be related to feather bacterial load in species under different evolutionary forces associated with migration and molt strategies.

The microbial load in feathers may also vary due to differences in body size. Previous studies have documented positive associations between parasite/protozoan abundance and animal body size/mass,^43^^,^^44^^,^^45^ supporting the macroecological theory of microbial species-area relationships^46^^,^^47^^,^^48^^,^^49^ and the simple isometric rule, i.e., that larger and heavier species have more body surface area and feather mass^2^ available for microbial colonization. This implies that heavier and structurally larger passerine species should have more feather area available for bacterial colonization, and their feather bacterial load should increase. Furthermore, larger species or individuals that are relatively heavier than conspecifics have been documented to increase their exploratory, risk-taking, and dispersal behavior,^50^^,^^51^^,^^52^ thereby altering the frequency and intensity of their contact with various environmental substrates/microbes and shaping their gut/skin microbiomes.^53^^,^^54^ Taken together, while the feather microbiome has been shown to vary with habitat and among individuals,^7^^,^^8^^,^^55^^,^^56^ it is unclear how the feather bacterial load is shaped by species’ life-history traits, such as migratory status, molt strategy, body mass, or preen gland volume.

**Figure 1:**
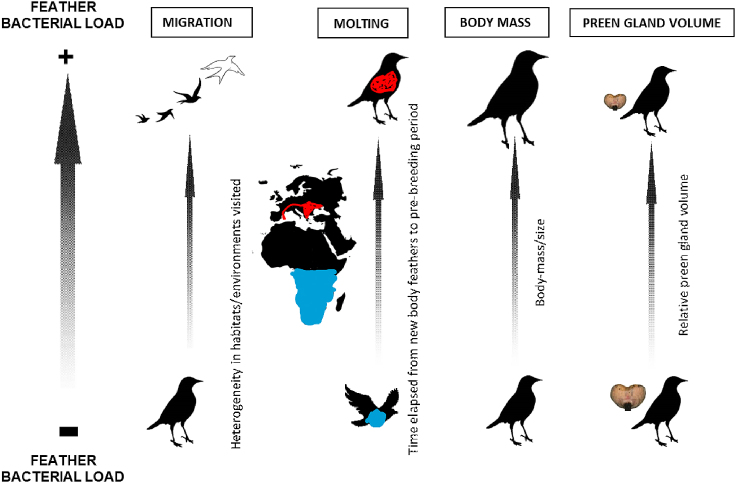
Schematic representation of postulated hypotheses MIGRATION: heterogeneity of habitats/environments visited increases in Palearctic long-distance migrants (flying flock silhouette, on the top) compared to Palearctic residents (standing bird silhouette, at the bottom). MOLTING: the duration of plumage contact with environmental microorganisms is higher in species that molt complete body feathers in breeding areas—“breeding molt strategy” (red)—compared to species that molt complete body feathers in non-breeding areas—“non-breeding molt strategy” (blue). BODY MASS: larger/heavier species/individuals have (1) more feather area/mass available for bacterial colonization and (2) more intense exploratory, risk-taking, and dispersal behavior, resulting in more intense feather bacterial loads due to increased contact with environmental substrates/microbes or conspecifics. PREEN GLAND VOLUME: individuals with larger preen glands are expected to preen their feathers more resulting in a reduction of feather bacterial loads.

**Figure 2:**
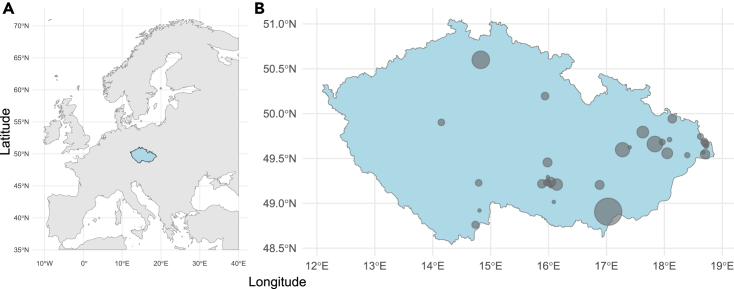
Palearctic passerines sampling region and sites (A) Sampling region (in blue, left); (B) geographical distribution of individual sampling sites in the Czech Republic (dots; right). Sample sizes, expressed as dots of variable size, ranged from 1 to 105 individuals sampled at a sampling site.

This study investigates the associations between migration and molt strategy, as well as body mass and preen gland volume, and the total and viable feather bacterial load during the pre-breeding period in Palearctic passerines. Based on the current knowledge, we hypothesize the following. (1) Migratory birds will have higher bacterial loads on their feathers than residents prior to breeding due to seasonal movements across geographic regions (i.e., increased heterogeneity of habitats visited) and due to the reduced capacity of preening during migration. (2) Feathers will accumulate microorganisms over time, which means that the duration of plumage contact with environmental microorganisms will be higher in species that undergo complete post-breeding molt of body feathers in breeding areas—“breeding molt strategy”—compared to species that undergo complete post-breeding molt in non-breeding areas—“non-breeding molt strategy.” Thus, the timing of molt will affect feather bacterial loads. (3) Feather bacterial loads will be higher in larger-bodied individuals and lower in individuals with larger preen gland volumes (postulated hypotheses are summarized graphically in Figure 1, and sampling region and locations of Palearctic passerines in Figure 2).

## Results

### Role of migration, molt strategy, body mass, and preen gland volume on the total and viable feather bacterial load in Palearctic passerines

**Figure 3:**
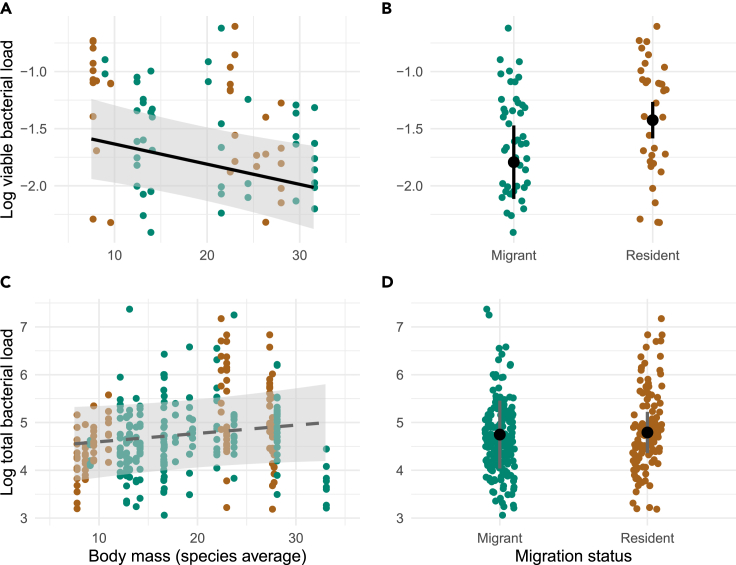
Relationships between viable and total feather bacterial load, interspecific variability in body mass, and migration status of Palearctic passerines (A and B) Relationships between log-transformed viable feather bacterial loads (ratio of viable to total feather bacterial cell counts per mg of contour body feathers) in Palearctic passerines (*n*_individuals_ = 79, *n*_species_ = 19) and (A) species average body mass (LMM: *p* = 0.008; solid line) and (B) migration status (LMM: *p* = 0.043). (C and D) Relationship between log-transformed total feather bacterial loads (16S rDNA gene copies per mg of contour body feathers) in Palearctic passerines (*n*_individuals_ = 316, *n*_species_ = 24) and (C) species average body mass (LMM: *p* = 0.213, dashed line) and (D) migration status (LMM: *p* = 0.831). Long-distance migrants are depicted with green dots, and resident species with brown dots. Lines and large dots depict mean ± SEM model fit. Error bars and polygons represent 95% confidence intervals.

Viable bacterial load in feathers was negatively related to average species body mass (linear mixed-effect model [LMM]: slope = −0.02, SE = 0.01; t = −2.7, *p* = 0.008; Table 1 and Figure 3A) and was slightly higher for residents (LMM: slope = 0.38, SE = 0.18; t = 2.1; *p* = 0.043; Table 1 and Figure 3B) but was not related to molting strategy (LMM: t = 1.34; *p* = 0.184; Table 1) or relative preen gland volume (LMM: t < 0.01, *p* = 0.99; Table 1). In contrast, none of the predictors considered explained the variation in total bacterial load in contour body feathers: species body mass (t = 1.29, *p* = 0.213, Table 2 and Figure 3C), migration status (t = 0.15, *p* = 0.882; Table 2 and Figure 3D), molting strategy (t = 0.27; *p* = 0.789; Table 2), or relative preen gland volume (t = 0.39; *p* = 0.695; Table 2). Within-species differences in body mass did not explain the variation in viable bacterial loads (t = −0.20; *p* = 0.839; Table 2) or total bacterial load (t = 1.13; *p* = 0.256; Table 1) in contour body feathers.

**Table 1:** Summary of linear mixed-effect model estimating relationships between tested predictors and viable bacterial loads in contour body feathers of Palearctic passerines sampled during the pre-breeding period

*Predictor*	*F*	*p*
Migration status^a^	4.25	0.043
Molt strategy	1.80	0.184
Body mass (within-species)	0.04	0.839
Body mass (among-species)^a^**∗**	7.47	0.008
Relative preen gland volume	<0.01	0.999

a Significant predictors (*p* < 0.05).

**Table 2:** Summary of linear mixed-effect model estimating relationships between tested predictors and total bacterial loads in contour body feathers of Palearctic passerines sampled during the pre-breeding period

*Predictor*	*F*	*p*
Migration status	0.01	0.905
Molt strategy	0.05	0.831
Body mass (within-species)	1.92	0.167
Body mass (among-species)	1.66	0.213
Relative preen gland volume	0.02	0.896

**Figure 4:**
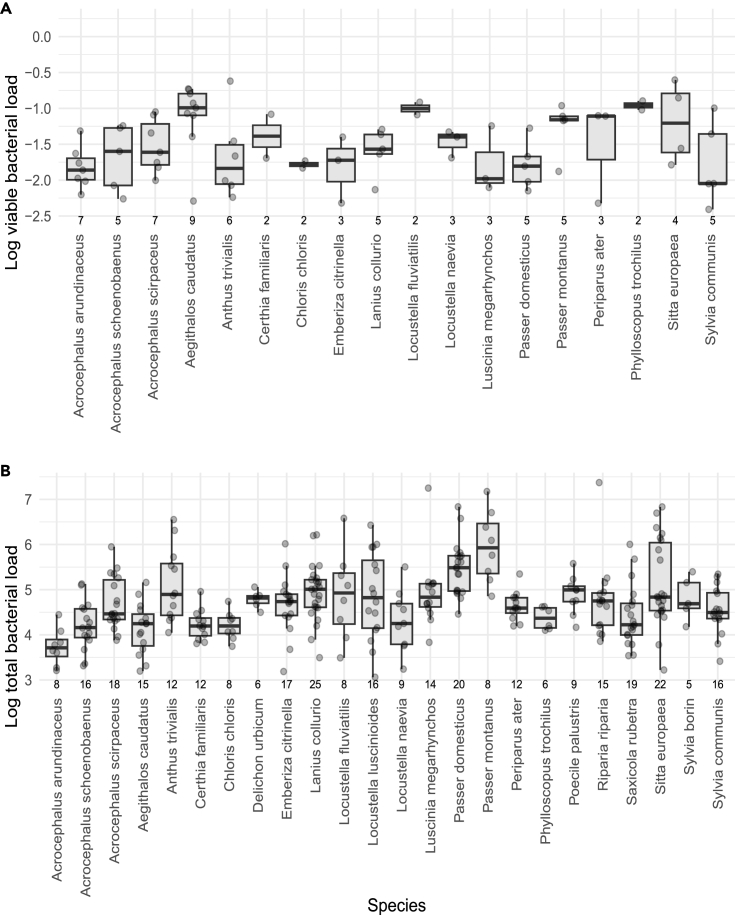
Interspecific variability in viable and total feather bacterial load in contour body feathers of Palearctic passerines (A) Interspecific variability of viable bacterial load (log-transformed ratio of viable to total bacterial cell counts per mg of feathers). (B) Interspecific variability in total bacterial load (log-transformed 16S rDNA gene copies per mg of contour body feathers). Boxplots express median value, 1st and 3rd quartiles, and maximum and minimum values. The numbers below each box represent the sample size.

Fixed effects explained 14% of the variation in viable bacterial load and 3% of the variation in total bacterial load. Random intercepts of species and sampling site combined explained 13% of the variation in viable bacterial load with species explaining 5% of the variation (see also Figure 4A for interspecific variability in viable feather bacterial load) and sampling site explaining 12% of the variation in viable bacterial load. Random intercepts of species, sampling, and year combined explained 34% of the variation in total bacterial load. Explained variation in total bacterial load for each random intercept was as follows: species = 26% (see also Figure 4B for interspecific variability in total feather bacterial load), site = 8%, and year = 6%.

### Intensity of viable and total feather bacterial load in Palearctic passerines

The mean viable bacterial load (i.e., the ratio of live to total bacterial cells in 1 mg of contour body feathers measured by flow cytometry) was 0.030 (mean; SD = 0.054; min = 0.004, max = 0.249). The mean viable bacterial load in resident species was 0.039 (mean; SD = 0.062, min = 0.005, max = 0.249), whereas in migratory species it was only 0.025 (mean; SD = 0.045, min = 0.004, max = 0.240). Flow cytometry bacterial counts in contour body feathers ranged from 1.14 × 10^3^ to 2.35 × 10^7^ (cells × mg^−1^). On average, the total bacterial load in contour body feathers (16S rDNA gene copies × mg^−1^) of Palearctic passerines was 53 × 10^3^ (mean, SD = 2 × 10^6^, min = 1 × 10^3^, max = 24 × 10^6^). While resident species had a mean total bacterial load in feathers (16S rDNA gene copies × mg^−1^) of 69 × 10^3^ (mean; SD = 2 × 10^6^; min = 2 × 10^3^; max = 15 × 10^6^), migratory species had a mean total bacterial load in feathers (16S rDNA gene copies × mg^−1^) of 45 × 10^3^ (mean; SD = 2 × 10^6^, min = 1 × 10^3^, max = 24 × 10^6^).

## Discussion

In this study, we investigated the associations of life-history characteristics, in particular, migration, molt strategies, body mass, and preen gland volume, with the two indices of bacterial load (total and viable) in the feathers of Palearctic passerines. We found that viable bacterial loads in contour body feathers were associated with between-species body mass variation, with heavier species having lower viable bacterial loads in their feathers compared to lighter species. In contrast, total bacterial load in feathers was not associated with either between- or within-species variation in body mass. These results did not support our hypotheses based on the macroecological theory of microbial species-area relationships^46^^,^^47^^,^^57^ and the simple isometric rule, i.e., that larger and heavier species have more body surface area and feather mass^2^ available for microbial colonization, or the results of previous studies documenting positive associations of protozoan/parasite abundance with animal body size.^43^^,^^44^^,^^45^ Instead, several alternative hypotheses and possible mechanisms may explain the observed association between viable bacterial load in feathers and between-species variation in body mass. First, there is strong evidence for associations between body mass and animal behavior^58^ including exploratory or risk-taking behavior,^50^^,^^51^^,^^52^ the intensity of which has been documented to alter the skin microbiome^54^^,^^59^ or parasite load.^60^ It is therefore likely that behavioral adaptations related to body mass are responsible for the observed associations between species’ body mass and viable bacterial load in feathers. Second, preening, which involves coating the feathers with sebaceous preen gland secretions, is an energetically costly activity^34^^,^^35^ that compromises immunity^15^ but is thought to reduce the bacterial load on the feathers.^19^ We can, therefore, assume that individuals that reduce their preening effort will suffer from a higher microbial load on the feathers but will benefit from higher energy reserves and immune capacity, which may be of great importance especially close to the breeding season. We suggest that the lower viable bacterial load in feathers of heavier and larger species in our study may be related to the assumption that larger species/individuals are better able to maintain a positive energy budget^61^^,^^62^ and maintain optimal preening activities without compromising their body/energy reserves compared to lighter and smaller species. Third, although so far documented only in amphibians, maintenance of stable resident skin microbiome highly resistant to microbial colonization from outside is dependent on metabolically costly production of skin antimicrobial proteins (AMPs),^63^^,^^64^ thus, larger-bodied individuals/species may invest more into the production of AMPs that can maintain lower viable bacterial load in feathers compared to smaller and lighter species. However, due to the limited research on the ultimate and proximate mechanisms explaining the relationship between body mass and vertebrate integumentary microbiomes, these alternative hypotheses need to be further tested in future studies.

The total bacterial load in the feathers of Palearctic passerines did not differ between residents and long-distance migrants, although the viable bacterial load was higher in residents compared to migrants. While previous studies have shown a clear effect of migration on the diversity and composition of feather microbiota,^27^^,^^28^ it appears that different host species-related mechanisms are involved in shaping feather microbial loads in passerines. Similarly, the molting strategies, which determine the exposure of body feathers to environmental microorganisms, did not play a role in feather bacterial loads in our study. This is consistent with the only study to document no effect of molt on feather bacterial load in mallards.^65^ However, we found that variation in total bacterial load was substantially explained by species identity (26% of explained variation; Figure 4B), sampling location (8% of explained variation), and year (6% of explained variation), suggesting that these factors overrule the effects of seasonal movement across geographical regions or timing of molt in shaping total feather bacterial load in Palearctic passerines. This is consistent with a previous study showing the important role of sampling location and species identity in determining bacterial and fungal loads on the flight feathers of 24 bird species.^14^ Similarly, previous comparative studies have found a primary effect of both host species and horizontal acquisition of microbes from the environment in shaping the diversity and composition of the feather microbiome.^7^^,^^8^ Furthermore, strongly species-specific and highly specialized feather-degrading and bacteriocin-producing microbial communities have been found in passerine body feathers,^7^ suggesting that host species-related factors are primary players in shaping the passerine feather microbiome. It is therefore worth studying further the between-species differences in the feather microbiome, including its functional properties.

Finally, in our study, we found no relationship between relative preen gland volume and total or viable bacterial load on feathers. This is in line with the results of previous studies, which did not find an association between preen gland size and total feather bacterial load.^37^^,^^39^^,^^40^^,^^41^ We also hypothesize that the potential of the preen gland to reduce feather bacterial load may be more related to inter- and intra-specifically varying chemical compounds in preen gland secretions^66^^,^^67^^,^^68^ rather than preen gland size. Furthermore, the antimicrobial potential of preen secretions to reduce feather bacterial load in birds is still controversial, with studies finding no antimicrobial activity,^69^ only *in vitro* antimicrobial activity against specific feather bacteria,^70^^,^^71^^,^^72^ while studies attempting to demonstrate broad-spectrum antimicrobial activity of preen gland secretions on total bacterial load in free-living birds have found no significant effect.^38^^,^^73^

The values of bacterial cell densities in feathers determined by flow cytometry in our study were approximately in the same order of magnitude as those found in previous studies on passerine birds.^36^^,^^74^^,^^75^^,^^76^ However, it should be noted that a direct comparison with these studies cannot be made because most of the previous studies using flow cytometry expressed the density of bacterial cells per feather, not per mg of feather weight as in our study. Similarly, the total bacterial load in our study was measured molecularly (qPCR) and expressed as the number of 16S rDNA gene copies per mg feather mass, which does not correspond to the number of bacterial cells due to the high variability of 16S rRNA gene in bacteria.^77^ A comparison with the only available comparative study that measured bacterial load in feathers of passerines by qPCR^14^ is also not possible because in that study bacterial load was expressed as ng of DNA per mg feather mass.

### Conclusions

To conclude, our findings indicate that the influence of stochastic ecological factors linked to the variation in avian life-history strategies across time and space on feather bacterial loads is negligible; rather, factors related to species, ecological niches they occupy, or individual characteristics contribute more to shaping the bacterial loads on feathers of Palearctic passerines prior to the breeding season.

### Limitations of the study

The limitations of this study are that it examines feather bacterial load only in passerine birds during pre-breeding season. Although passerines are one of the most diverse groups of extant birds,^78^ further extension of the feather microbiome studies to other groups of birds would certainly be of interest. It is also known that reproduction is an energetically demanding process, forcing parent individuals to make trade-offs between investments in reproduction, immunity, feather growth, and self-maintenance.^35^^,^^38^^,^^79^ Therefore, studying the characteristics of the feather microbiome during different annual life-history stages (e.g., breeding and/or early post-breeding periods) with respect to different breeding strategies (e.g., cavity and/or open-cup nesting) and sex could provide very valuable insights into the role of microorganisms in the evolution of life-history strategies in birds. Finally, this study focuses exclusively on the feather bacterial load, which has been documented as a key feature of the feather microbiome associated with feather quality, immunity, or reproductive success.^15^^,^^19^ However, focusing future studies on microbial diversity and functional metagenomics may be of great interest, providing a useful perspective on avian host-microbiome interactions.

## Resource availability

### Lead contact

Further requests for information or resources should be directed to and will be fulfilled by the lead contact, Veronika Gvoždíková Javůrková, (veronika.javurkova@gmail.com).

### Materials availability

This study did not generate new unique reagents or materials.

### Data and code availability

• The dataset and customized R script code are deposited at Zenodo data repository and are publicly available at Zenodo: https://doi.org/10.5281/zenodo.10410326. DOIs are also listed in the key resources table.
• Sample metadata are stored and available for reuse in the AviSample Network database: https://avisample.net/ under the following identifiers: AS05235–AS05326; Brlík et al. 2022). Accession link and identifiers are available also in the key resources table.
• Any additional information required to reanalyze the data reported in this paper is available from the lead contact upon request.

## Acknowledgments

This work and V.G.J. were supported by the 10.13039/501100003246Dutch Research Council (NWO – Nederlandse Organisatie voor Wetenschappelijk Onderzoek) project number OCENW.KLEIN.541 of the research program Open Competition Domain Science – KLEIN, the 10.13039/501100001824Czech Science Foundation project 14-16861P, and Institutional Research Support of the Czech Academy of Sciences RVO: 68081766. V.B. was supported by 10.13039/100007397Charles University (UNCE/24/SCI/006). We thank Eva Holánová and Radka Valterová for their help with the lab work and sample sorting and processing; Jason Dean, Markéta Kyptová, Marko Dachev, and Jozef Janda for their assistance with flow cytometry analyses; and Daniel Křenek, Kateřina Ševčíková, Jiří Brožek, Jindřich Sedláček, Jiří Porkert, Jaroslav Koleček, Michal Šulc, and Václav Jelínek for their assistance with bird trapping and feather samples collection.

## Author contributions

V.G.J. (corresponding author): conceptualization, resources, funding acquisition, methodology, supervision, investigation, data curation, project administration, visualization, and writing – original draft; V.B. (corresponding author): conceptualization, investigation, data curation, formal analysis, visualization, and writing – original draft; P.P.: conceptualization, investigation, and writing – review and editing; M.P. and P.H.: investigation and writing – review and editing; M.W.D.: writing – review and editing; B.I.T. and J.F.S.: supervision, funding acquisition, and writing – review and editing.

## Declaration of interests

The authors declare no competing interests.

## STAR★Methods

### Key resources table

**Table:** 

REAGENT or RESOURCE	SOURCE	IDENTIFIER
**Chemicals, peptides, and recombinant proteins**		

Maximum Recovery Diluent - MRD	Sigma Aldrich	Cat. no.: 07233-500G-F
Phosphate buffered saline – PBS sterile	Sigma Aldrich	Cat. no.: P2272
Phosphate buffered saline – PBS tablets	Sigma Aldrich	Cat. no.: P4417
Tryptic Soy Agar - TSA	Sigma Aldrich	Cat. no.: 22091
S.O.C. medium	Thermo Fisher Scientific	Cat. no.: 15544034
Tween 80	Sigma Aldrich	Cat. no.: P8074
PEG - polyethylene glycol 6000	Sigma Aldrich	Cat. no.: 89510
Sodium deoxycholate	Sigma Aldrich	Cat. no.:30970
20 mM Tris buffer	Sigma Aldrich	Cat. no.: PPB022
EDTA - Ethylenediaminetetraacetic acid	Sigma Aldrich	Cat. no.: E6758
NaCl – Sodium chloride	Sigma Aldrich	Cat. no.: S9625

**Critical commercial assays**		

RTP® Bacteria DNA Mini Kit	STRATEC Molecular	Cat. no.:1033200300
KAPA SYBR® FAST for LightCycler®480 Mastermix	Roche (Merck)	Cat. no: KK4610
Microbial DNA-Free Water	Qiagen	Cat. no: 338132
gBlock® 1555 bp fragment of *Bacillus subtilis* 16S rRNA (reference sequence accession number NR_102783.1, GenBank).	Javurkova et al.^80^ IDT Integrated DNA Technologies	N/A - customized order at: https://sg.idtdna.com/pages/products/genes-and-gene-fragments/double-stranded-dna-fragments/gblocks-gene-fragments
BD™ Cell Viability Kit	BD Biosciences	Cat. no: 349483
BD™ Liquid Counting Beads	BD Biosciences	Cat. no: 335925

**Deposited data**		

Raw data	This paper	Zenodo: https://doi.org/10.5281/zenodo.10410326
Sample metadata	This paper	AviSample Network database: https://avisample.net/) under the following identifiers: AS05235–AS05326
R codes for final analysis	This paper	Zenodo: https://doi.org/10.5281/zenodo.10410326

**Oligonucleotides**		

16S rRNA primers:	Javurkova et al.^80^	N/A
27F: AGAGTTTGATCCTGGCTCAG		
534R: ATTACCGCGGCTGCTGG		

**Software and algorithms**		

R 4.1.2	The R Project for Statistical Computing	https://www.r-project.org/
CytExpert software	Beckman Coulter	https://www.beckman.pt/flow-cytometry/research-flow-cytometers/cytoflex/software
LightCycler® 480 System software	Roche	https://lifescience.roche.com/global/en/products/product-category/lightcycler.html

### Experimental model and study participant details

In this study, we captured and sampled for chest contour body feathers 316 free-living adult individuals of 9 residents and 15 long-distance migratory passerines at 29 sites in the Czech Republic during March-May 2015-2016 (Table S1 and Figure 2). We captured 316 individuals (226 males and 62 females) in their pre-breeding period. Sex was not determined for 28 individuals of monomorphic species due to unavailability of source genetic material for molecular sexing. Complete information on the species identity, sex, age, morphometric parameters and geographic site of sampled birds can be found in metadata files on Zenodo data repository and AviSample Network database (DOIs and accession identifiers available in the key resources table).

The study was approved by the Ethical Committee of the Central Commission for Animal Welfare of the Ministry of Agriculture of the Czech Republic under the permit 47941/ENV/15–2247/630/15 and carried out in accordance with Directive 2010/63/EU for animal experiments (https://www.eurofawc.com/home/16).

### Method details

#### Bird sampling

Sampled individuals were captured using mist-nests in the pre-breeding phase to minimize confounding effects of seasonal change in feather microbial load.^27^^,^^75^^,^^76^ From each individual, we collected 15–20 chest contour feathers using sterile forceps prior to any manipulation in mist-nest to avoid contamination. The contour feathers were placed into dry sterile 1.8mL cryotubes (Thermo Scientific - Nunc) and stored in a cooler at 5°C for <6 h. Cryotubes were then stored at −20°C until the analysis of bacterial load.

After contour feather sampling, morphometric data (i.e., body weight and preen gland width, length and height) were taken with a precision of 0.01 mm using digital calipers (Proma WT4171). Preen gland measures were then used to calculate relative preen gland volume, as proxy of secretory potential, following the equation introduced in previous studies.^42^^,^^81^ Sex of sampled individuals was determined either visually in the field based on sexual dichromatism and dimorphism^82^ or CHD gene molecular markers using DNA extracted from feather samples.^83^ All individuals were released back into the wild after sampling.

#### Assignment of moult strategies

We classified each species into one of two groups based on the timing and extent of their body feather moult, following the method outlined in previous complex study.^84^ We identified two distinct moult strategies that lead to differences in the exposure of body feathers to environmental microorganisms before breeding. Species that moult their contour body feathers at their breeding grounds during the post-breeding period were categorized as having a “Breeding moult strategy”. In contrast, species that moult during the non-breeding period were classified as having a “Non-breeding moult strategy”. Consequently, the duration of exposure of feathers to the environmental microorganisms prior to our sampling during the pre-breeding stage was long for the “Breeding moult strategy” and short for the “Non-breeding moult strategy” (see also Figure 1).

#### Quantification of feather bacterial loads

##### qPCR quantification of total bacterial load

###### DNA extraction

We used protocol similar to our previous study.^80^ Screw-capped cryotubes containing feather samples were removed from the freezer and feathers were transferred to extraction tubes of the RTP Bacteria DNA Mini Kit (STRATEC Molecular GmbH, Berlin, Germany) using sterile forceps. Genomic DNA was then extracted according to Protocol 5 of the isolation kit (Isolation of microbial DNA from tissue biopsies). All sample manipulations were randomised and isolation steps were performed by one person (VGJ) under sterile conditions in a laminar flow hood in a non-invasive laboratory. The weights of individual feather samples used for DNA extraction were determined after DNA isolation as follows. Immediately after DNA extraction, each feather sample was washed three times with 1 mL of distilled water to remove residual lysis buffer and dried at 95°C in a ThermoMixer C (Eppendorf, Hamburg, Germany). The dry feather mass of each sample was then measured to an accuracy of 0.01 mg using a laboratory balance (KERN, ABT 120-5DM). The initial feather mass of each sample was then used to calculate the total 16S rDNA gene copies per mg of contour body feathers.

###### qPCR assay

Universal Eubacteria primer set 27F (5′-AGAGTTTGATCCTGGCTCAG -3′) and 534R (5′- ATTACCGCGGCTGCTGG -3′) and KAPA SYBR FAST for LightCycler480 Mastermix (Sigma Aldrich) were used for qPCR amplification on the LightCycler 480 System intrument. The qPCR reaction (15 μL) contained 7.5 μL KAPA SYBR FAST for LightCycler480 Mastermix, 6.02 μL Microbial DNA-Free Water (Qiagen, USA), 0.24 μL of each primer at a concentration of 10 μM (i.e., final concentration for each primer was 160 nM) and 1 μL DNA template. Each qPCR reaction was performed in triplicate on a 96-well Hard-Shell 480 PCR plate (CLR/WHT) (Bio-Rad, USA). The plate was sealed with Microseal C film (Bio-Rad, USA). Amplification conditions were as follows: one pre-incubation cycle at 95°C for 3 min followed by 40 amplification cycles at 95°C for 10 s, 50°C for 25 s and 72°C for 1 s.

To determine the specificity of the amplification, gel electrophoresis of the amplified PCR products was performed together with analysis of the melting curves of the products. A melting curve was obtained by slow heating at 2.2°C/step from 65°C to 97°C with fluorescence collection at 0.5°C intervals.

###### Quantification of 16S rDNA copy numbers

As the known 16S rRNA copy number variation of *B. subtilis* is intermediate compared to other bacteria,^77^ and *B. subtilis* and *Bacillus* spp. are abundant in feathers,^85^^,^^86^ we reduced potential under/overestimation of 16S rDNA gene copies in our samples using the gBlock 16S rRNA fragment of *B. subtilis* as a standard. The synthetized lyophilized gBlock 1555 bp fragment (NCBI Integrated DNA Technologies, USA) of *B. subtilis* 16S rRNA (reference sequence accession number NR_102783.1, GenBank) was diluted with sterile filtered TE buffer to a concentration of 10 ng/μL, which corresponded to 5.868 × 10^9^ 16S rDNA gene copies. A dilution series of gBlock was then generated ranging from 5.68 × 10^3^ to 5.68 × 10^7^ of 16S rDNA gene copies and used as a standard. Using the master calibration strategy,^87^^,^^88^ a single master calibration curve with efficiency, slope and amplification factor of 95.1%, −3.45 and 1.94, respectively, was generated from four independent gBlock dilution series. This calibration curve, which showed a close correlation between Cp and actual 16S rDNA gene copies in the gBlock standard dilutions (R^2^ = 0.976), was used to quantify the total bacterial load on feathers, which was related to the initial feather mass and expressed as the number of 16S rDNA copies per mg of feathers. LightCycler 480 System software was used for qPCR data analysis.

###### Flow cytometry quantification of viable feather bacterial load

We quantified viable feather bacterial load using flow cytometry based on differential staining of dead and all bacterial cells with propidium iodide (PI) and thiazole orange (TO) using the BD Cell Viability Kit (BD Biosciences, USA) in a subset of the dataset (*n* = 79) containing dry-frozen feather samples collected only in 2016 and therefore with identical freezing duration (≈7 months) to standardize the potential effects of freezing on bacterial cell viability, which mainly include osmotic imbalance in cells and formation of intracellular ice crystals.^89^^,^^90^ As both of these cell viability impairments are enhanced by the presence of water,^91^ (i.e., mostly typical for cultured bacterial cells preserved in nutrient broth or buffer) and the dehydration process (i.e., water reduction) strongly eliminates both of these viability affecting factors,^90^ we snap-frozen dry feather samples without adding any additional water-rich buffer. Therefore, we believe that although some viability reduction occurred, it was minimised and all samples analyzed were equally affected by maintaining an identical freezing duration of ≈7 months for all samples analyzed by flow cytometry.

Due to an optimization pre-experimental process during which we tested various feather bacteria releasing methods (see Method S1 in Supplemental information for detailed methods description), we were able to quantify viable bacterial cells both freely persistent as well as attached on contour feathers^76^ per each feather sample.

###### Feather bacterial cell suspension preparation and staining procedures

Slowly-thawed feather samples were placed in 2 mL of sterile filtered Maximum Recovery Diluent (MRD; 07233, Sigma Aldrich) and incubated for 1 h, vortexed for 1 min at 2500 rpm (IKA MS3 digital) and sonicated for 30 s using a BANDELIN SONOPULS HD 2070 ultrasonic homogeniser with MS73 sonotrode under the following conditions: cycle = 7; power amplitude = 20%. Samples were cooled in a water bath during sonication. After sonication, the feathers samples in MRD were vortexed again for 1 min at 2500 rpm and then passed through the sterile 50μm mesh CellTricks filter (Sysmex Partec GmbH, Germany) into the 2mL sterile plastic tube (Eppendorf) to reduce the amount of cell debris in the suspension.

Tubes containing 2 mL of bacterial cell suspension were again centrifuged at 5000 rpm for 5 min and 1700 μL of supernatant was aspirated. Then 500 μL of sterile filtered PBS (Sigma Aldrich) was added, vortexed for 30 s and centrifuged again at 5000 rpm for 5 min. 450 μL of supernatant was carefully aspirated and the remaining cell suspension was vortexed for 20 s, stained by adding 3.5 μL of TO and 3.5 μL of PI provided in BD Cell Viability Kit (BD Biosciences, NJ) and incubated for 12min in the dark. Together with feather bacterial samples, four control samples containing a 1:1 ratio of live/dead cultured bacterial suspension were prepared as follows. 900 μL of cultured bacterial suspension in PBS and 900 μL of suspension containing dead bacteria (i.e., cultured bacteria killed by 96% ethanol) in PBS were mixed and divided into 4 tubes of 350 μL each. PI control was prepared by adding 3.5 μL of PI only, TO control by adding 3.5 μL of TO only, PI + TO control by adding 3.5 μL of TO and 3.5 μL of PI and NO DYES control was left without TO or PI dyes.

Stained feather and control bacterial cell suspensions were processed using an Apogee A50 Micro cytometer (Apogee Flow Systems) with the following settings: sample volume = 150 μL; sample flow rate = 0.75 (μL/min); sheath (pressure) = 150 mbar. Prior to each measurement, BD Liquid Counting Beads (BD Biosciences, San Jose, CA), a flow cytometry bead standard, was used to accurately quantify the live, dead and total bacterial counts in a sample. Analysis of cytometry data was performed using CytExpert software (Beckman Coulter). All counts were controlled for initial dry feather mass, weighed after sample processing and expressed as bacterial cells per 1 mg of contour body feathers. Finally, we quantified viable bacterial load (i.e., the ratio of viable bacterial cell counts to total bacterial cell counts in the 1 mg of feather sample).

### Quantification and statistical analysis

#### Phylogenetic signal analysis

We assessed the phylogenetic signal in both response variables (i.e., total and viable bacterial load). We quantified maximum credibility phylogenetic relationships between the species using maxCladeCred function from phangorn R package^92^ using 100 Hackett backbone phylogenetic trees randomly sampled from https://birdtree.org.^93^ For fitting models accounting for phylogenetic relationships between species, we used Markov chain Monte Carlo generalized linear mixed models from the R package MCMCglmm^94^ with parameter expanded (V = 1, nu = 1, alpha.mu = 0) weakly-informed inverse-Wishart priors for random effects. Each model was run with 1,000,000 iterations, with a burn-in of 200,000 and thinning of 100 to ensure effective sample sizes exceeding 8,000 for all model parameters.

In total, we fit two models with log-transformed total and viable bacterial loads as responses without fixed effects (i.e., null models) and random effects of phylogeny, species identity and sampling site. We quantified the phylogenetic signal (λ) in the model as Variance(phylogeny)/(Variance(phylogeny) + Variance(residual)). Prior specification was following: prior.ratio1 <- list(R = list(V = diag(1), nu = 0.002), G = list(G1 = list(V = diag(1), nu = 1, alpha.mu = 0, alpha.V = diag(1)∗1000), G2 = list(V = diag(1), nu = 1, alpha.mu = 0, alpha.V = diag(1)∗1000), G3 = list(V = diag(1), nu = 1, alpha.mu = 0, alpha.V = diag(1)∗1000))).

Log viable bacterial load (posterior mode and 95% credible intervals) resulted in phylogenetic signal λ = 0.003 (<0.001; 0.418) and log total bacterial load (posterior mode and 95% credible intervals) in phylogenetic signal λ = 0.001 (<0.001; 0.477). As the resulting λ was <0.003 in both cases, we did not include phylogeny in our main models to reduce complexity, given the sample sizes.

#### Statistical analysis

We tested associations between two response variables – total bacterial load (i.e., the 16S rDNA gene copies per 1 mg of contour body feathers) and viable bacterial load (i.e., the proportion of viable bacterial cells to total bacterial flow cytometry cell counts in 1 mg of feather sample) and a set of predictors using linear mixed-effects models (LMMs). As fixed effect predictors we considered migration status, moulting strategy, average species body mass, within-species variation in body mass (i.e., body mass measurements standardized within each species) and relative preen gland volume. To identify relationships between these variables, we log-transformed the response variables and fit two linear mixed-effects models while accounting for the data structure by including random intercepts of species, sampling site and year for total bacterial load (only total bacterial loads collected over two years) and random intercepts of species and sampling site for viable bacterial load (only samples from one year have been used – see above). We did not consider sex as a predictor of viable and total bacterial loads in order to keep our models parsimonious due to: i) unbalanced sex ratios between species (total of 62 females and 226 males) and ii) lack of differences in bacterial loads between females and males (viable bacterial loads: F = 0.01, df = 71, *p* = 0.902; total bacterial loads: F = 1.14, df = 268, *p* = 0.287) after running univariate linear mixed effects models (LMMs) with sex as the only predictor and the random effects structure used in the main models (see above).

The two main models differed in sample size due to analytical processes detailed above (n _total bacterial load_ = 316; n _viable bacterial load_ = 79). We used lmer function from *lme4* R package to fit LMMs.^95^ Model assumptions were checked by assessing normality of DHARMa residuals^96^ and multicollinearity of predictors checked by variance inflation factors (VIFs <3.1 for all explanatory variables; Figure S1). Marginal and conditional R^2^ were calculated using r.squaredGLMM function using R package *MuMIn*.^97^ All analyses were performed in R 4.1.2.^98^

## Supplemental information

Document S1. Figures S1, Table S1, and Method S1
