# Supplementary material for: Host traits rather than migration and molting strategies explain feather bacterial load in Palearctic passerines
Source: iScience. 2024 Sep 30;27(11):111079. doi: 10.1016/j.isci.2024.111079 (PMC11513523; doi:10.1016/j.isci.2024.111079)

iScience, Volume *27*

Supplemental information

Host traits rather than migration and molting strategies explain feather bacterial load in Palearctic passerines

Veronika Gvoždíková Javůrková, Vojtěch Brlík, Petr Heneberg, Milica Požgayová, Petr Procházka, Maurine W. Dietz, Joana Falcao Salles, and B. Irene Tieleman

SUPPLEMENTAL INFORMATION

Supplemental information summary content:

Table S1 – Complete overview of Palearctic passerine species sampled in this study for analysis of total and viable feather bacterial load related to STAR Methods and Figure 2.

Method S1 - Pre-experimental optimization of viable feather bacterial load flow cytometry quantification related to STAR Methods

Figure S1 - VIFs (Variance Inflation Factors) expressing the absence of strong collinearity between fixed predictors in LMMs (Linear Mixed Effect Models) associated with STAR Methods

Table S1. Complete overview of Palearctic passerine species sampled in this study for analysis of total and viable feather bacterial load related to STAR Methods and Figure 2. List of Palearctic passerine species used in this study for analysis of A) total bacterial load and B) viable bacterial load with information on number of samples per species, migration status and moult strategy, type of analysis, number of sampling localities and years sampled. The full dataset is available in the Zenodo data repository: https://zenodo.org/doi/10.5281/zenodo.10410326.

A) TOTAL BACTERIAL LOAD - qPCR METHOD

Species	Samples	Migration status	Moulting strategy	Localities	Years
Acrocephalus arundinaceus	8	Long-distance migrant	Non-breeding	1	3
Acrocephalus scirpaceus	18	Long-distance migrant	Non-breeding	1	2
Acrocephalus schoenobaenus	16	Long-distance migrant	Non-breeding	1	2
Aegithalos caudatus	15	Resident	Breeding	4	2
Anthus trivialis	12	Long-distance migrant	Non-breeding	4	1
Certhia familiaris	12	Resident	Breeding	4	2
Delichon urbicum	6	Long-distance migrant	Non-breeding	1	1
Emberiza citrinella	17	Resident	Breeding	6	2
Carduelis chloris	8	Resident	Breeding	3	2
Lanius collurio	25	Long-distance migrant	Non-breeding	4	2
Locustella fluviatilis	8	Long-distance migrant	Non-breeding	3	2
Locustella luscinioides	16	Long-distance migrant	Non-breeding	1	2
Locustella naevia	9	Long-distance migrant	Non-breeding	2	3
Luscinia megarhynchos	14	Long-distance migrant	Breeding	1	2
Passer domesticus	20	Resident	Breeding	3	2
Passer montanus	8	Resident	Breeding	2	1
Periparus ater	12	Resident	Breeding	3	2
Phylloscopus trochilus	6	Long-distance migrant	Non-breeding	3	2
Poecile palustris	9	Resident	Breeding	4	2
Riparia riparia	15	Long-distance migrant	Non-breeding	3	1
Saxicola rubetra	19	Long-distance migrant	Non-breeding	1	1
Sitta europea	22	Resident	Breeding	9	2
Sylvia borin	5	Long-distance migrant	Non-breeding	4	2
Sylvia communis	16	Long-distance migrant	Non-breeding	6	2

B) VIABLE BACTERIAL LOAD - FLOW CYTOMETRY METHOD

Species	Samples	Migration status	Moulting strategy	Localities	Years
Acrocephalus arundinaceus	7	Long-distance migrant	Non-breeding	1	1
Acrocephalus scirpaceus	7	Long-distance migrant	Non-breeding	1	1
Acrocephalus schoenobaenus	5	Long-distance migrant	Non-breeding	1	1
Aegithalos caudatus	9	Resident	Breeding	3	1
Anthus trivialis	6	Long-distance migrant	Non-breeding	2	1
Certhia familiaris	2	Resident	Breeding	1	1
Emberiza citrinella	3	Resident	Breeding	1	1
Chloris chloris	2	Resident	Breeding	1	1
Lanius collurio	5	Long-distance migrant	Non-breeding	1	1
Locustella fluviatilis	2	Long-distance migrant	Non-breeding	1	1
Locustella luscinioides	1	Long-distance migrant	Non-breeding	1	1
Locustella naevia	3	Long-distance migrant	Non-breeding	2	1
Luscinia megarhynchos	3	Long-distance migrant	Breeding	1	1
Passer domesticus	5	Resident	Breeding	2	1
Passer montanus	5	Resident	Breeding	2	1
Periparus ater	3	Resident	Breeding	1	1
Phylloscopus trochilus	2	Migrant	Non-breeding	2	1
Sitta europaea	4	Resident	Breeding	2	1
Sylvia communis	5	Long-distance migrant	Non-breeding	3	1

Method S1 – **Pre-*experimental optimization of viable feather bacterial load flow cytometry quantification***

<u>*Pre-experimental optimization of releasing bacterial cells from feathers*</u>

As bacterial cells have been documented to be present on feathers in two forms as "free" and "attached",[1-3] we tested i) various sonication treatments, ii) 9 different buffers, iii) a combination of selected buffers + sonication and vortex treatment to optimise the method for releasing bacteria from feathers with minimal loose of bacterial cell viability.

<u>*Testing of the effect of sonication on the viability of bacterial cells*</u>

Although mechanical treatment such as sonication is necessary to release bacteria attached to the feather structure,[1,3] sonication has been shown to affect bacterial cell viability.[4] Therefore, we first aimed to test the effect of different intensities of sonication on bacterial cell viability. To do this, we used bacterial cultures grown on TSA (tryptic soy agar) for 48 hours at 38°C from extra feathers plucked from free-living birds. Bacterial cells were then harvested from the TSA plates and diluted in S.O.C. medium (Thermo Fisher Scientific, 15544034) at 0.5 McFarland turbidity standard, equivalent to 1.5 x 10^8 colony forming units (CFU/ml), to prepare a mixed bacterial cell suspension for viability testing. We then prepared 16 sterile 10mL tubes with 5 mL aliquots of the mixed cell suspension and treated each tube with different intensities (amplitudes) and durations of sonication using a BANDELIN SONOPULS HD 2070 ultrasonic homogeniser with MS73 sonotrode (Table 1). Each tube was cooled on ice during sonication.

Table 1. Sonication intensities measured as amplitude setting and duration (seconds) applied to each separate tube of mixed viable bacterial cultures.

Tube	Amplitude (sonication)	Duration (sec.)
1	20%	10
2	20%	30
3	20%	60
4	20%	180
5	30%	10
6	30%	30
7	30%	60
8	30%	180
9	70%	10
10	70%	30
11	70%	60
12	70%	180
13	90%	10
14	90%	30
15	90%	60
16	90%	180

The proportion of viable/dead bacterial cells was then measured using the following method. First, the bacterial suspension was passed through a sterile 50µm mesh CellTricks filter (Sysmex Partec GmbH, Germany) into a 5 mL sterile plastic tube (Eppendorf) to reduce the amount of cell debris in the suspension. The tubes containing the bacterial cell suspension were again centrifuged at 5000 rpm for 5 minutes and the supernatant was aspirated. The cell suspension was then stained according to the recommendations and protocol of the BD™ Cell Viability Kit (BD Biosciences, NJ). Along with the treated bacterial suspension, three control samples containing untreated bacterial suspension, a 1:1 ratio of live/dead cultured bacterial suspension, and suspension containing dead bacteria (i.e., cultured bacteria killed by 96% ethanol). Stained treated and control bacterial cell suspensions were processed using an Apogee A50 microcytometer (Apogee Flow Systems) with the following settings: sample volume = 150 µL; sample flow rate = 0.75 (µL/min); sheath (pressure) = 150 mbar. Based on this pre-experiment, we found that sonication at 20% amplitude power for 30 seconds maintained the highest proportion of viable bacterial cells in suspension (proportion of dead bacterial cells ranged between 11-15%) compared to other sonication treatments.

Testing of different feather bacterial cells releasing buffers

In order to most effectively release bacterial cells from the feather while maintaining maximum cell viability,[4-6] we tested 9 different buffers containing compounds documented to effectively release bacteria from biofilms on different matrices. Specifically, we used 1) PBS buffer containing 1 PBS

tablet (phosphate buffered saline manufactured by Sigma Aldrich, P4417) dissolved in 200 mL deionised water; 2) Buffer 2 - containing 0.85% NaCl + 0.1% Tween 80 in ddH2O; 3) Buffer 3 - 0.05% Tween 80 in PBS; 4) Buffer 4 - 2.5% PEG 6000 (polyethylene glycol 6000) in PBS; 5) Buffer 5 - 0.5% PEG 6000 + 0.1% sodium deoxycholate in PBS; 6) Buffer 6 - 20 mM Tris buffer; 7) Buffer 7 - 0.3 mM EDTA in PBS; 8) Buffer 8 - 1 mM EDTA in PBS; 9) Maximum Recovery Diluent - MRD (07233, Sigma Aldrich). All buffers were syringe filtered prior to use using sterile 0.2µM PES membrane syringe filters, 25 mm diameter (Nalgene, 725-2520).

Feathers plucked from several individuals of free-living species were then mixed, divided into 32 15 mL sterile tubes and treated with different release buffers, sonication for 30 s at 20% amplitude and including testing of the vortexing step (yes/no) for 1 min at 2500 rpm. All samples were then prepared for staining as described above and the efficacy and viability of the bacterial cells released from the feathers were measured. MRD (Maximum Recovery Diluent) was found to be the best buffer, releasing the highest number of bacterial cells per mg of feathers. For further analysis of the viable bacterial load of feathers, we therefore used MRD and sonication for 30 seconds at 20% amplitude and vortexing for 1 minute (see "*Feather bacterial cell suspension preparation and staining procedures* "section of the STAR Methods).

Supplemental references:

1. Saag, P., Kilgas, P., Magi, M., Tilgar, V., and Mand, R. (2012). Inter-annual and body topographic consistency in the plumage bacterial load of Great Tits. J. Field Ornithol. *83*, 94-100. 10.1111/j.1557-9263.2011.00359.x.
2. Saag, P., Mand, R., Tilgar, V., Kilgas, P., Magi, M., and Rasmann, E. (2011). Plumage bacterial load is related to species, sex, biometrics and fledging success in co-occurring cavity-breeding passerines. Acta Ornithol. *46*, 191-201. 10.3161/000164511x62596.
3. Saag, P., Tilgar, V., Mand, R., Kilgas, P., and Magi, M. (2011). Plumage Bacterial Assemblages in a Breeding Wild Passerine: Relationships with Ecological Factors and Body Condition. Microb. Ecol. *61*, 740-749. 10.1007/s00248-010-9789-0.
4. Brandl, M.T., and Huynh, S. (2014). Effect of the surfactant Tween 80 on the detachment and dispersal of *Salmonella enterica* serovar Thompson single cells and aggregates from Cilantro leaves as revealed by image analysis. Appl. Environ. Microbiol. *80*, 5037-5042. 10.1128/aem.00795-14.
5. Lutskiy, M.Y., Avneri-Katz, S., Zhu, N., Itsko, M., Ronen, Z., Arnusch, C.J., and Kasher, R. (2015). A microbiology-based assay for quantification of bacterial early stage biofilm formation on reverse-osmosis and nanofiltration membranes. Separ. Purif. Technol. *141*, 214-220. 10.1016/j.seppur.2014.12.003.
6. Bredholt, S., Maukonen, J., Kujanpää, K., Alanko, T., Olofson, U., Husmark, U., Sjöberg, A.M., and Wirtanen, G. (1999). Microbial methods for assessment of cleaning and disinfection of food-processing surfaces cleaned in a low-pressure system. Eur. Food Res. Technol. *209*, 145-152. 10.1007/s002170050474.

Figure S1 - VIFs (Variance Inflation Factors) expressing the absence of strong collinearity between fixed predictors in LMMs (Linear Mixed Effect Models) associated with STAR Methods

Variance Inflation Factors (VIF) bar chart showing no strong collinearity (VIF < 3.1 for all predictors) among fixed predictors for individual Linear Mixed Effect Models (LMMs) testing A) viable feather bacterial load and B) total feather bacterial load

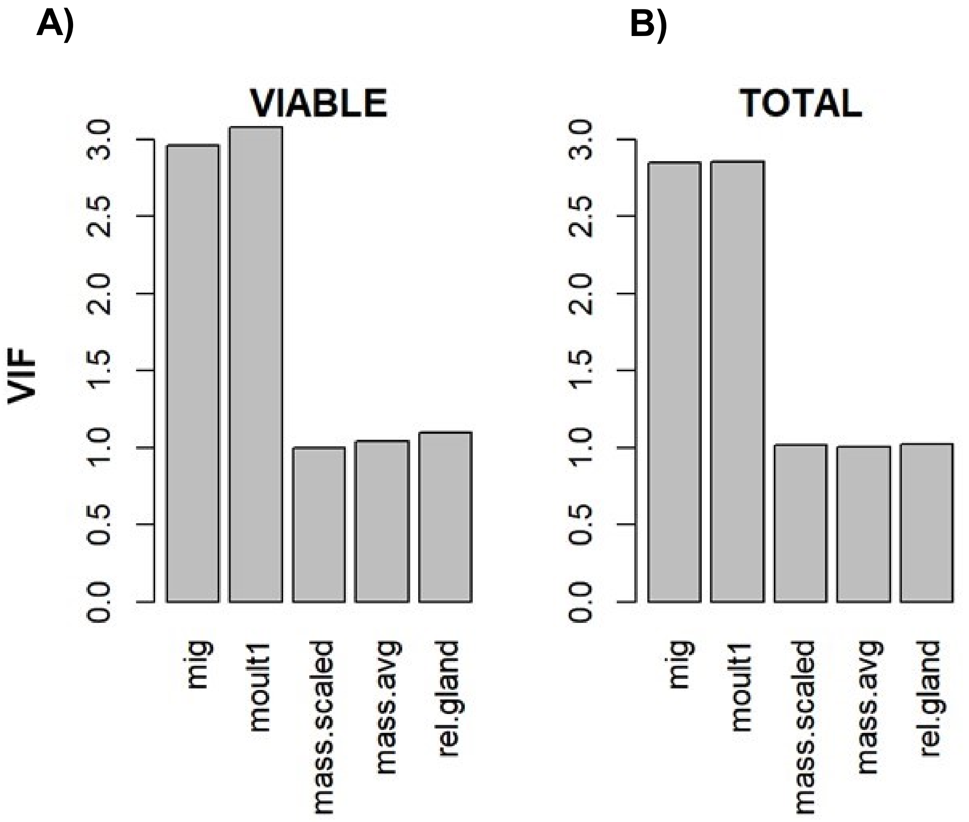

Supplement: Document S1. Figures S1, Table S1, and Method S1 [file mmc1.pdf]
